# 胸腺肿瘤微创手术研究进展

**DOI:** 10.3779/j.issn.1009-3419.2018.04.06

**Published:** 2018-04-20

**Authors:** 文涛 方, 志涛 谷, 克能 陈

**Affiliations:** 1 200030 上海，上海交通大学附属胸科医院胸外科 Department of Thoracic Surgery, Shanghai Chest Hospital, Shanghai Jiaotong University, Shanghai 200030, China; 2 100142 北京，北京大学肿瘤医院胸外科 Department of Thoracic Surgery, Beijing Cancer Hospital, Beijing 100142, China

**Keywords:** 胸腺肿瘤, 微创手术, 胸腔镜手术, Thymic malignance, Minimally invasive surgery, Video-assisted thoracoscopic surgery

## Abstract

手术是胸腺肿瘤最重要的治疗方式。近年来，胸腺肿瘤的微创手术在国内外获得迅速推广。微创手术目前主要用于早期胸腺肿瘤的外科治疗，已有证据表明，微创手术可有效减少手术创伤、有效加快患者的术后恢复以及降低术后疼痛，并且目前已有一定临床证据初步证实其肿瘤学效果与开放手术相当。同时随着外科技术的不断进步，在保证外科学和肿瘤学原则的前提下，微创手术有可能使包括局部进展期、诱导治疗后以及复发转移的患者从中获益。

## Abstract (en)

Surgery is the most important therapy for thymic malignances. The last decade has seen increasing adoption of minimally invasive surgery (MIS) for thymectomy. MIS for early stage thymoma patients has been shown to yield similar oncological results while being helpful in minimize surgical trauma, improving postoperative recovery, and reduce incisional pain. Meanwhile, With the advance in surgical techniques, the patients with locally advanced thymic tumors, preoperative induction therapies or recurrent diseases, may also benefit from MIS in selected cases.

目前认为所有胸腺肿瘤都具有恶性潜能^[1]^，即使A型胸腺瘤也可存在远处转移，而早期胸腺瘤完全切除后亦可能出现复发。胸腺肿瘤是在胸部实体肿瘤中相对罕见^[2]^，同时又是相对惰性的肿瘤，即便疾病进展或复发后仍有可能获得长期生存，极难开展大规模的前瞻性随机研究以获得高质量证据来指导临床实践。因此对胸腺肿瘤的诊治尚存在诸多争议。近年来通过全球性国际合作[国际胸腺肿瘤协会（International Thymic Malignancy Interest Group, ITMIG）]和包括中国在内的[中国胸腺肿瘤研究协作组（Chinese Allience for Research in Thymomas, ChART）]各地区各国家的区域性合作^[3]^，围绕这些问题进行的临床研究获得了较好的结果，使胸腺肿瘤的诊治逐步从单纯依赖个人经验开始走上循证医学的轨道。

目前手术仍然是胸腺肿瘤最重要的治疗方式，通过根治性切除最有望获得治愈^[4]^。近年来胸腺外科进展迅速，突出体现在电视辅助胸腔镜手术（video-assisted thoracoscopic surgery, VATS）、机器人辅助手术等微创外科技术获得迅速推广，然而临床指南目前并不推荐微创手术作为首选术式，原因是缺乏有力度的临床研究证据支持^[5]^。

要确立微创手术在胸腺肿瘤外科治疗中的作用和地位，首先需要证实其安全可行，且相比于胸骨正中劈开等开放术式创伤更小、有利于患者恢复。这方面已有大量临床研究报道，证据力度已经较为充实。例如上海市胸科医院多年前即已报道^[6]^相比于胸骨正中切口开放手术而言，胸腔镜胸腺切除手术时间更短、术中出血量更少，患者恢复更快因而术后重症加强护理病房（intensive care unit, ICU）和住院时间也明显缩短。Ruckert等^[7]^报道腔镜胸腺手术后早期患者疼痛显著较胸骨正中切口减轻因而需要使用镇痛药物更少时间更短，而且腔镜手术对患者的肺功能影响也显著减少，腔镜和开放患者术后即刻肺功能减低分别为35%和65%，腔镜患者术后第3天肺功能即完全恢复而开放手术患者仅恢复55%。Hess等^[8]^对20项相关临床报道中2, 068例手术患者进行的荟萃分析也显示相比于开放手术而言微创胸腺手术失血量更少、术后引流量减少、术后住院时间缩短。

相比于围术期结果，更为重要的是鉴于手术治疗的对象是恶性肿瘤，必须证实微创手术的肿瘤学效果至少不亚于开放手术。国际上微创手术21世纪后方开始逐步使用于胸腺肿瘤的外科治疗^[9]^，ChART回顾性数据库也显示2010年后Masaoka Ⅰ期和Ⅱ期肿瘤微创手术的比例已攀升至40%以上，在部分中心甚至接近100%^[10]^。但是由于胸腺肿瘤大多恶性程度较低、发展相对缓慢，往往需要比其他恶性肿瘤更长的随访时间（推荐10年生存率指标及相对于生存率来说更能反映肿瘤治疗结果的复发转移率）来评价治疗效果，因此直至最近才开始出现对微创手术肿瘤学长期随访结果的研究报道。

ITMIG回顾性数据库研究通过倾向匹配分析从全球2, 514例胸腺瘤患者中筛选出266对微创和开发手术病例进行比较，结果发现在去除肿瘤大小、分期和组织学类型的影响后，两组手术根治性切除率完全一样，均达到96%；多因素分析显示R0切除率与手术径路并不相关，手术年份推移（代表外科技术的进步）、肿瘤分期（分期越高切除率越低）、胸腺切除范围（全胸腺切除更易获得根治）才是独立的预测因素^[11]^。鉴于以往绝大多数研究都提示胸腺肿瘤根治性切除是和临床病理分期、组织学类型并列的三大独立预后因素^[4]^，ITMIG的这项研究事实上通过发现微创胸腺切除术与开放手术具有相似的肿瘤切除率间接地证实了微创手术的肿瘤学效果。

日本胸腺肿瘤联盟（Japanese Association of the Research on the Thymus, JART）最近发表回顾性研究^[12]^，通过倾向性匹配从日本32家医院2, 835例Masaoka Ⅰ期-Ⅱ期胸腺瘤患者中挑选出140对腔镜和正中切开的病例进行比较，结果腔镜组和开放组分别仅3例和1例患者切缘阳性（R1）；两组术后5年总体生存率相似，分别为97.9%和97.1%；更重要的是无复发生存率无显著差异，分别为93.9%和95%。值得注意的是该研究未纳入胸腺癌患者，且同样由于微创手术开展较晚，腔镜组中位随访时间（3.7年）显著短于开放组（5.2年）。

中国胸腺肿瘤研究协作组对于微创胸腺手术的价值也进行了详细的研究。Wang等^[10]^首先利用ChART数据库中1, 117例Masaoka-Koga Ⅰ期-Ⅱ期胸腺肿瘤患者临床资料进行回顾性分析，发现3例术后30天死亡患者均接受开放手术，腔镜组无一发生。腔镜组总体手术切除率（98.8%）和5年无病生存率（92%）均显著高于开放组（88.7%和83%），而两组5年总体生存率相似，均为92%；术后病理证实为Masaoka-Koga Ⅰ期-Ⅱ期患者的复发率亦相似，分别为3.3%和4.7%。鉴于两组在手术切除范围、肿瘤大小、临床病理分期和组织学类型上存在明显差异，多因素分析表明仅肿瘤分期及其世界卫生组织（World Health Organization, WHO）组织学类型是决定长期生存的独立预后因素，而与手术径路无关。

由于之前的研究使用的是术前临床分期作为入组条件，且未能很好地消除混杂因素的影响，Gu等^[13]^进一步利用ChART数据库中1, 087例UICC Ⅰ期（相当于Masaoka Ⅰ期-Ⅱ期）胸腺肿瘤病例，通过倾向匹配分析获得腔镜和开放各110例患者，两组之间在肿瘤大小、病理分期、组织学类型和术后辅助治疗上均匹配良好，没有差异。结果腔镜组和开放组中位随访时间分别为26个月和36个月，两组术后总体生存率（85.7% *vs* 93.1%, *P*=0.539）、无病生存率（92.5% *vs* 91.9%, *P*=0.773）和累积复发率（7.1% *vs* 5.8%, *P*=0.522）均无显著差异，合并肌无力患者症状改善率亦相似（83.3% *vs* 88.2%, *P*=0.589），再次证实微创胸腺手术可获得与开放手术相似的远期疗效。

需要指出的是，尽管上述国际性和地区性合作通过大病例组研究、多因素分析和倾向匹配等多种统计学方法试图获得更为科学的结果，但仍然无法避免回顾性研究和非随机对照的内在缺陷和混杂偏倚，而且总体随访时间仍然偏短。鉴于胸腺肿瘤的较为少见和相对惰性，几无可能开展前瞻性随机对照临床研究，目前唯有通过延长随访时间、提高随访精度来进一步比较微创和开放手术的长期肿瘤学效果。

经典的胸腺肿瘤手术治疗方式是经胸骨正中切口行全胸腺切除术，因为胸骨劈开后前纵隔暴露良好，对于没有外侵的早期肿瘤切除胸腺在外科技术上并不增加困难，可以保证手术切除的根治性，而且胸腺对于成年人来说已经基本丧失了原有的免疫功能，切除胸腺不会造成患者的功能损失。随着微创胸腺手术的开展，这一观念开始受到挑战，目前的胸腔镜、机器人等微创手术大多经一侧胸腔入路，与开放手术的前外侧或后外侧开胸入路相似，此种入路对于暴露胸腺上极和对侧胸腺有一定困难，虽然在腔镜视野和器械的帮助下可以达到和正中切口相似的切除范围，但同样存在一定的操作难度和无名静脉意外损伤造成出血和中转的风险。近年来有报道腔镜下行部分胸腺切除可能获得与全胸腺切除相似的效果^[14-16]^，这些研究都出自亚洲国家，估计与亚洲医生对腔镜技术接受更快以及对一侧胸腔入路行胸腺部分切除更有经验有关。Fang等^[9]^利用ITMIG回顾性数据库对亚、欧、美三大洲手术径路进行了比较，发现在1430例Masaoka-Koga Ⅰ期-Ⅱ期胸腺肿瘤患者中亚洲腔镜等微创手术的比例超过30%，远远高于北美（15.9%）和欧洲（9.6%），同时亚洲病例中胸腺部分切除的比例（31.7%）同样远远高于北美（5.4%）和欧洲（2.4%），多因素分析表明除地域影响外微创手术或外侧开胸径路是胸腺部分切除的独立相关因素。另一个更重要的问题是上述3个回顾性研究都是单中心小病例组报道，且随访时间较短，对于相对恶性程度较低的胸腺瘤而言难以证实部分胸腺切除的肿瘤学效果。

事实上中国胸腺肿瘤研究协作组的多中心回顾性研究结果更好地说明了这个问题^[17]^。在ChART数据库1, 047例Masasoka-Koga Ⅰ期-Ⅱ期胸腺肿瘤中有近1/4的患者接受了部分胸腺切除，胸骨正中切口多为全胸腺切除，外侧开胸切开多为部分胸腺切除，但腔镜等微创手术全胸腺与部分胸腺的比例相当，说明中国医生目前微创手术还是以全胸腺切除为主。对随访结果的多因素分析表明，两种切除范围10年总体生存率相同（90.9% *vs* 89.4%），总体复发率两组也相似（3.1% *vs* 5.4%）；然而进一步的分层分析显示对于Masaoka-Koga Ⅰ期肿瘤两种术式的复发率没有统计学差异（3.2% *vs* 1.4%），但是在Masaoka-Koga Ⅱ期患者中胸腺部分切除后的复发率显著高于全胸腺切除（14.5% *vs* 2.9%, *P*=0.001）。鉴于Masaoka-Koga Ⅰ期（包膜完整）和Ⅱ期肿瘤（显微镜下包膜浸润或纵隔脂肪局部侵犯）无论是术前影像学检查还是术中肉眼观察均无法区别，加之胸腺肿瘤还存在多原发或多病灶的可能性，所以以目前的证据来看无论开放还是微创手术均应遵循外科学解剖切除和肿瘤学根治性切除的原则，推荐行全胸腺切除以保证手术治疗的效果。

总的来说，微创胸腺手术目前主要被用于早期肿瘤的外科治疗，指Masaoka-Koga Ⅰ期-Ⅱ期或相对应的国际抗癌联盟（Union for International Cancer Control, UICC）Ⅰ期肿瘤，但从外科技术来看侵犯心包局部或邻近肺组织局限性受侵的部分Masaoka-Koga Ⅲ期或UICC Ⅱ期-Ⅲa期肿瘤^[18]^腔镜下切除并不困难，同样可以达到和开放手术相似的切除彻底程度。Fang等^[19]^在2017年世界肺癌大会上报道115例UICC Ⅱ期-Ⅲa期肿瘤的外科治疗结果，通过1:2的倾向匹配分析后26例腔镜和52例开放手术患者的R0切除率均达到77%，通过术后35个月的中位随访期发现腔镜可以获得和开放手术相当的肿瘤学效果，总体生存率分别为100%和95.2%，3年累积复发率分别为0.052和0.167，均无统计学差异；而相比于开放手术而言，接受腔镜手术的患者术中出血量显著减少（127 mL *vs* 219 mL, *P*=0.005）、术后胸管引流时间[(3±1.2) d *vs* (5±4.7) d, *P*=0.005]和总体住院时间[(5.9±3.1) d *vs* (9.6±5.1) d, *P* < 0.001]均显著缩短，体现了微创手术的优越性。

随着微创外科技术的不断进步，不仅是局部外侵周围结构的UICC Ⅲa期肿瘤，对于部分复发转移患者以及肿瘤外侵严重但经过诱导治疗后获得降期的病例通过腔镜手术也有可能获得彻底切除的可能性^[20]^，而对于这些需要多种方式综合治疗的患者腔镜技术更能发挥微创手术的优势，通过减少手术创伤、加快功能恢复，帮助减降低围术期风险，使患者能更好地耐受术后辅助治疗，以达到期望的肿瘤学效果。

综上所述，微创手术技术在胸腺肿瘤的外科治疗方面已相当成熟，通过减少手术创伤能够有效帮助患者的恢复、降低功能损失，目前也已经有一定临床证据初步证实其肿瘤学效果，但仍有待长期随访进一步验证。同时随着外科技术的不断进步，在保证外科学和肿瘤学原则的前提下，微创手术的使用范围已不局限于早期胸腺肿瘤，有可能使包括局部进展期、诱导治疗后以及复发转移患者从中获益。

**Table:** 中国胸腺肿瘤协作组成员名单

魏煜程	青岛大学附属医院	刘永煜	辽宁肿瘤医院
李印	郑州大学附属肿瘤医院	方文涛	上海交通大学附属胸科医院
陈克能	北京大学肿瘤医院	谷志涛	上海交通大学附属胸科医院
陈和忠	长海医院	韩泳涛	四川省肿瘤医院
崔友斌	吉林大学第一附属医院	傅剑华	中山大学肿瘤防治中心胸科
张仁泉	安徽医科大学第一附属医院	于振涛	天津医科大学肿瘤医院食管肿瘤科
丁建勇	复旦大学附属中山医院	张鹏	天津医科大学总医院
陈岗	复旦大学附属中山医院	王允	四川大学华西医院
陈椿	福建医科大学附属协和医院	周鑫明	浙江肿瘤医院
庞烈文	复旦大学附属华山医院	朱成楚	浙江省台州医院
柳阳春	江西省人民医院		
